# Metabolic Engineering of *Yarrowia lipolytica* for Conversion of Waste Cooking Oil into Omega-3 Eicosapentaenoic Acid

**DOI:** 10.1021/acsengineeringau.4c00053

**Published:** 2025-02-13

**Authors:** Jiansong Qin, Na Liu, Umer Abid, Sarah M. Coleman, Yongdan Wang, Qiang Fu, Seongkyu Yoon, Hal S. Alper, Dongming Xie

**Affiliations:** † Department of Chemical Engineering, University of Massachusetts Lowell, Lowell, Massachusetts 01854, United States; ‡ McKetta Department of Chemical Engineering, The University of Texas at Austin, Austin, Texas 78712, United States

**Keywords:** omega-3 fatty acids, eicosapentaenoic acid, triglycerides, waste cooking oil, *Yarrowia lipolytica*

## Abstract

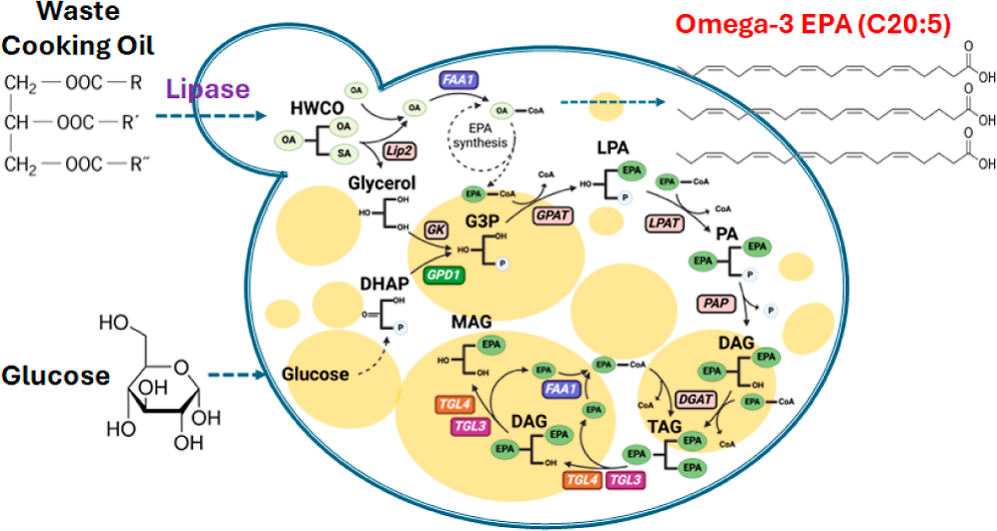

Omega-3 polyunsaturated fatty acids (PUFAs), especially eicosapentaenoic acid (EPA, C20:5), are crucial dietary fats known for their numerous health benefits. However, traditional sources of EPA, like fish oil, raise sustainability and environmental concerns, underscoring the need for alternative production methods. The engineered oleaginous yeast *Yarrowia lipolytica* has emerged as a promising candidate for sustainable production of EPA. This study explores the efficient production of EPA with an earlier engineered*Y. lipolytica* strain Y8412, utilizing waste cooking oil (WCO) as an alternative carbon source. While cofeeding WCO resulted in increased total lipid content, it also caused an increase in intracellular free fatty acid (FFA) levels, which can be toxic to cells and reduce EPA synthesis. To solve this issue, we first overexpressed *FAA1* and *GPD1* genes converting excess FFAs into triglycerides (TAGs). Additionally, we knocked out *TGL3*/*4* genes, which encode lipases linked to lipid bodies, to minimize the degradation of TAGs back into FFAs. The modified strains significantly reduced intracellular FFA levels and improved EPA production. Notably, the *TGL4* knockout strain Y8412T4^–^ showed 57% increase in EPA production titer and nearly 50% increase in carbon conversion yield compared to the parental strain Y8412 fed with glucose only. These findings suggest that preventing TAG degradation by knocking out *TGL4* is an effective approach for enhanced EPA production when WCO is used to partially replace glucose as the carbon source. This study offers an effective engineering strategy for low-cost, high-yield, and sustainable production of omega-3 fatty acids from waste feedstocks.

## 1 Introduction

Omega-3 PUFAs, specifically EPA and docosahexaenoic acid (DHA, C22:6) are essential dietary lipids renowned for their extensive health-promoting properties.^1,2^ These fatty acids are integrated into various tissues throughout the body, particularly within the phospholipid bilayer of cell membranes, where they influence membrane fluidity and support anti-inflammatory mechanisms.^3^ EPA and DHA are crucial for maintaining and supporting vital physiological processes across different stages of life. During fetal development, these omega-3 fatty acids play a pivotal role in the formation and maturation of the nervous system, including the development of the brain and eyes, ensuring proper cognitive and visual function after birth.^4^ DHA is a vital structural component of all cellular membranes and is highly concentrated in the brain and retina, underscoring its significance in neural and visual health.^5^ Furthermore, both EPA and DHA serve as precursors to a variety of bioactive lipid mediators, including resolvins, protectins, and maresins, which play a central role in regulating inflammation and promoting tissue repair.^6,7^ EPA and DHA-derived lipid mediators are increasingly recognized as therapeutic agents with the potential to address the underlying inflammatory processes of many chronic diseases, enhancing long-term health outcomes.^6^

Obtaining sufficient levels of EPA and DHA solely through dietary sources can be difficult, despite their natural production by aquatic plants like algae and their abundance in marine life. Algae serve as the foundational producers of these essential omega-3 fatty acids, which are then accumulated in the tissues of marine animals through the food chain.^8,9^ Among the various sources available, fish oil is widely regarded as the most concentrated and accessible natural provider of omega-3s, making it a popular choice for supplementation to meet daily nutritional needs.^10^ However, concerns about the sustainability of global fish stocks are mounting due to the severe depletion of marine populations caused by decades of overfishing. This overexploitation has raised alarms about the long-term availability of fish-derived omega-3 fatty acids.^11^ Additionally, environmental contamination of marine ecosystems has led to the accumulation of harmful substances in fish.^12^ Beyond these environmental and health issues, the reliance on fish oil for omega-3 production comes with further challenges, including high production costs, variable product quality, and significant uncertainties regarding its long-term ecological and economic sustainability.^12^ These factors highlight the urgent need to explore alternative, more sustainable sources of omega-3s. As high-purity EPA demonstrates superior therapeutic and preventive effects for certain diseases, there is a growing focus on optimizing its production process, achieving higher purity, and enhancing its bioavailability for maximum effectiveness.^13^

**Figure 1:**
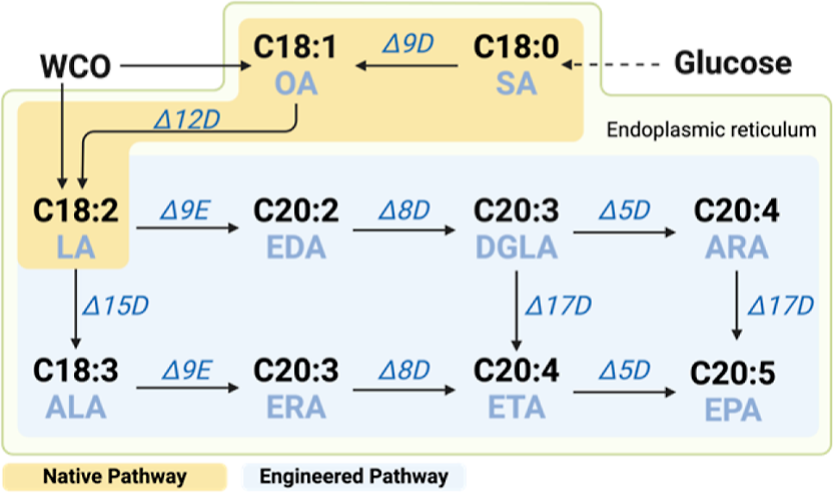
Metabolic pathways for production of omega-3 EPA (C20:5) from glucose and/or WCO. Solid arrows: one-step processing; dashed arrows: multistep metabolic pathways. In cytoplasm, glucose is converted into saturated fatty acids via glycolysis, TCA cycle, and fatty acid synthesis pathway. C16 ∼ C18 saturated fatty acids enter the endoplasmic reticulum for further desaturation and elongation through engineered pathways involving various desaturases and elongases. Major components of WCO include OA (C18:1) and LA (C18:2). Δ9D: Δ9 desaturase; Δ12D: Δ12 desaturase; Δ9E: Δ9 elongase; Δ8D: Δ8 desaturase; Δ5D: Δ5 desaturase; Δ17D: Δ17 desaturase; Δ15D: Δ15 desaturase. SA: stearic acid; OA: oleic acid; LA: linoleic acid; EDA: eicosadienoic acid; DGLA: dihomo-γ-linolenic acid; ARA: arachidonic acid; ALA: α-linolenic acid; ERA: eicosatrienoic acid; ETA: eicosatetraenoic acid; EPA: eicosapentaenoic acid.

Recently, the oleaginous yeast*Yarrowia lipolytica* has gained considerable interest as a promising cell factory for producing value-added biochemicals, including lipids, omega-3 fatty acids, citric acid, triacetic acid lactone (TAL), and carotenoids.^14−20^ Its natural capacity to accumulate significant lipid content (especially under nutrient-limited conditions), along with its genetic tractability, enables the efficient production of single-cell oils, biodiesel, and valuable omega-3s like EPA and DHA.^21^ A *Y. lipolytica* strain engineered by DuPont can produce EPA more than 50% of its total lipid content.^22,23^ The *Y. lipolytica* strain Y8412 (ATCC PTA-10026) generated by DuPont can produce omega-3 EPA from glucose, utilizing its native metabolic pathways.^23,24^ Through a series of desaturation and elongation steps, these omega-3 fatty acids are further converted into EPA, which constitutes 56% of the total fatty acid content. Additionally, the strain demonstrates a lipid concentration of 27% or more of its dry cell weight (DCW).^24^ The EPA production follows the biosynthetic pathway involving key intermediates palmitic acid (C16:0), stearic acid (SA, C18:0), oleic acid (OA, C18:1), and linoleic acid (LA, C18:2) along with the intermediates from engineered pathways (Figure 1) such as eicosadienoic acid (EDA, C20:2), dihomo-γ-linolenic acid (DGLA, C20:3), and eicosatetraenoic acid (ETA, C20:4), which are converted stepwise to produce EPA.^22,23^ Further development of a two-stage continuous fermentation led to significantly higher production EPA titers and rates.^25,26^ However, the current metabolic pathway for producing EPA from glucose still suffers challenges in low conversion yield, mainly caused by the low efficiency of fatty acid synthesis, which requires a sufficient supply of ATP and cofactor NADPH. Therefore, using a more energy-efficient carbon source from a low-cost feedstock to partially or completely replace glucose for EPA production should be considered, as discussed in our recent review.^27^

In this study, we investigated the use of WCO to partially replace glucose for enhanced EPA production. Figure 1 also highlights our proposed new pathway in*Y. lipolytica*that uses WCO as a direct carbon source of C16 ∼ C18 fatty acids, which can then be further converted into EPA via the engineered pathway.^28^ Currently, the global production of WCO is estimated approximately 42 million tons each year.^29,30^ When not disposed properly, WCO can lead to issues such as clogged drains and sewers, as well as contamination of water sources and soil.^30^ The use of WCO as a feedstock for biodiesel production is a well-established approach to repurposing this waste and reducing environmental impact.^31,32^ However, given the large quantities of WCO generated globally, more diverse applications are needed to address the associated environmental concerns effectively.^27,33,34^ Our proposed approach to convert WCO into EPA not only helps address the concern of waste material disposals but also provides a new route for sustainable biomanufacturing of similar high-value products with significant social and economic values.

The previously engineered *Y. lipolytica* Y8412, designed primarily for producing EPA from glucose, was used as a starting strain to investigate the EPA production from WCO. As the strain’s beta-oxidation was knocked out for enhanced lipid production,^24^ a cofeeding strategy was developed, which uses glucose as the primary carbon source for cell growth and energy balance and WCO as a second carbon source for enhanced lipid and EPA production. Subsequent modifications of genes including *FAA1*, *GPD1*, and *TGL3*/*4* that regulate the formation of free fatty acids (FFAs) and triacylglycerides (TAGs) were also studied to further improve EPA production efficiency.

## 2 Materials and Methods

### 2.1 Medium and Chemicals

YPD medium was prepared with 10 g/L yeast extract (VWR, USA), 20 g/L peptone (RPI, USA), and 20 g/L dextrose (VWR, USA). Luria–Bertani (LB) medium supplemented with 100 mg/L ampicillin (VWR, USA) contained 5 g/L yeast extract (VWR, USA), 10 g/L tryptone (RPI, USA), 10 g/L sodium chloride (Fisher Chemical, USA), and 100 mg/L ampicillin (VWR, USA). YPD hygromycin plates consisted of YPD medium, 15 g/L agar (Fisher Chemical, USA), and 500 mg/L hygromycin (RPI, USA). The YNB uracil drop-out medium included 1.67 g/L yeast nitrogen base without amino acids and ammonium sulfate (Becton, Dickinson and Company, USA), 5 g/L ammonium sulfate (Fisher Chemical, USA), and 0.94 g/L yeast synthetic drop-out medium supplements without uracil (Sigma-Aldrich, USA). YNB uracil drop-out plates contained YNB medium and 15 g/L agar. The 5-FOA plates were prepared using YNB uracil drop-out medium, 0.2 g/L 5-FOA (GoldBio, USA), and 12 mg/L uracil (Sigma-Aldrich, USA). The trace metal solution (100×) comprised 15 g/L citric acid, 1.5 g/L calcium chloride dihydrate, 10 g/L iron sulfate heptahydrate, 0.39 g/L zinc sulfate heptahydrate, 0.38 g/L copper sulfate pentahydrate, and 0.3 g/L manganese sulfate tetrahydrate. The fed-batch flask culture stock medium (5×) contained 12.5 g/L yeast extract, 5 g/L ammonium sulfate, 50 g/L potassium phosphate monobasic (Thermo Scientific, USA), 125 g/L potassium phosphate dibasic, 0.5 g/L magnesium sulfate (Sigma-Aldrich, USA), 2.5 mL/L trace metal solution (100×), 1.5 mg/L thiamine hydrochloride (Thermo Scientific, USA), and 250 g/L dextrose. The seed culture medium for fed-batch flask fermentation included 5 g/L yeast extract, 5 g/L ammonium sulfate, 6 g/L potassium phosphate monobasic, 2 g/L sodium phosphate dibasic, and 40 g/L dextrose. Waste cooking oil was collected from a local fast-food restaurant, which originated from cooking French fries in Canola oil. The lipase solution (2×) for hydrolyzing waste cooking oil contained 15 g/L lipase (Ward’s Science, USA), 7.1 g/L sodium phosphate dibasic, 3 g/L sodium phosphate monobasic, and 1 mL/L Triton X-100 (Fisher Bioreagent, USA).

### 2.2 Strain Engineering

The parental strain *Y. lipolytica* Y8412 was purchased from ATCC (no. PTA-10026). All other strains were developed from Y8412, as listed in Table 1. This study used the hygromycin-resistance gene (*Hyg*^R^) and the *URA3* gene as selection markers (Figures S1a and S2a). The constitutive promoter *EXP* and the TEF terminator were used to express the hygromycin-resistance gene and *URA3* gene (Figure S2a).

**Table 1:** Metabolically Engineered *Y. lipolytica* Strains Used in This Study

strain	genotype	reference
Y8412	ATCC20362, *ΔPex3*, P_Yat1_-ME3S-T_lip2_, P_GPD_-ME3S-T_Pex20_, P_FBAINm_-EgD9eS-T_lip2_, P_EXP1_-EgD9eS-T_lip1_, P_GPAT_-EgD9e-T_lip2_, P_Yat1_-EgD9eS-T_lip2_, P_FBAINm_-EgD8M-T_lip1_, P_GPD_-EaD8S-T_Pex16_, P_FBAIN_-EgD8M-T_lip1_, P_GPD_-EaD8S-T_Pex16_, P_Yat1_-E389D9eS/EgD8M-T_lip1_, P_GPD_-FmD12-T_Pex20_, P_Yat1_-FmD12-T_Oct_, P_EXP1_-FmD12S-T_Aco_, P_EXP1_-EgD5M-T_Pex16_, P_Yat1_-EaD5SM-T_Oct_, P_FBAIN_-EgD5SM-T_Pex20_, P_GPDIN_-EgD5SM-T_Aco_, P_GPM_-EgD5SM-T_Oct_, P_FBAINm_-PaD17-Tlip1, P_EXP1_-PaD17-T_Pex16_, P_Yat1_-PaD17S-T_lip1_, P_Yat1_-YICPT-T_Aco_, P_Yat1_-MCS-T_lip1_	Hong et al^24^
Y8412F^+^	Y8412, P_*Yat1*_-*FAA1*-T_*lip2*_-loxP-P_EXP_-*Hyg*^*R*^-T_TEF_-loxP	this study
Y8412G^+^	Y8412, P_*Yat1*_-*GPD1*-T_*lip2*_-loxP-P_*EXP*_-*Hyg*^*R*^-T_TEF_-loxP	this study
Y8412T3^–^	Y8412, *ΔTgl3*::loxP-P_EXP_-*Hyg*^*R*^-T_TEF_-loxP	this study
Y8412T4^–^	Y8412, *ΔTgl4*:: loxP-P_*EXP*_-*Hyg*^*R*^-T_TEF_-loxP	this study
Y8412T4^–^U^–^	Y8412, *ΔTgl4*:: loxP-P_EXP_-*Hyg*^*R*^-T_TEF_-loxP, *ΔUra3*	this study
Y8412T3^–^T4^–^	Y8412T4^–^U^–^, *ΔTgl3*:: loxP-P_*EXP*_-*Ura3*-T_TEF_-loxP	this study
Y8412F^+^T4^–^	Y8412T4^–^U^–^, P_*Yat1*_-*FAA1*-T_*lip2*_-loxP-P_*EXP*_-*Ura3*-T_TEF_-loxP	this study
Y8412G^+^T4^–^	Y8412T4^–^U^–^, P_*Yat1*_-*GPD1*-T_*lip2*_-loxP-P_EXP_-*Ura3*-T_TEF_-loxP	this study

#### 2.2.1 General Protocols

##### 2.2.1.1 Plasmid Construction

The plasmid pCfB6574 (Addgene #106132), which contains a *LIP2* terminator and a hygromycin-resistance gene expression cassette, was obtained from Addgene. The plasmid was linearized using the restriction enzyme AsiSI (New England Biolabs, USA) at 37 °C in a C1000 thermal cycler (Bio-Rad, USA). The *YAT1* promoter, *FAA1* (YALI0_D17864g) open reading frame (ORF), and *GPD1* (YALI0_B02948g) ORF were amplified from ATCC 20362 by PCR using Q5 High-Fidelity DNA Polymerase (New England Biolabs, USA) according to the manufacturer’s protocol (Figure S2a). Primers were designed following the instructions from the NEBuilder HiFi DNA Assembly kit (New England Biolabs, USA), ensuring that each PCR product had overlapping regions with adjacent fragments to facilitate the assembly process. The assembled DNA fragments were then transformed into 5-alpha competent *Escherichia coli* cells (New England Biolabs, USA) to finalize plasmid construction.

##### 2.2.1.2 Linear DNA Cassette Construction by Overlap Extension PCR

The two fragments to be ligated were amplified by PCR using primer sets with additional overhangs (Figure S1a and S2b). The overhang of the reverse primer of the upstream fragment overlapped with the extra overhang of the forward primer of the downstream fragment. In the second step, the two fragments were mixed and subjected to PCR without additional primers; the overlapping regions allowed the fragments to anneal and serve as primers and templates for each other. In the third step, the product from the second step (without purification) was used as a template for PCR amplification using the forward primer of the upstream fragment and the reverse primer of the downstream fragment to amplify the ligated fragments.

##### 2.2.1.3 Yeast Transformation

Yeast cells were grown in 10 mL of YPD medium in a 125 mL flat-bottom Erlenmeyer flask overnight until the OD_600_ reached 1.5. The culture was centrifuged at 2000 rpm for 10 min. After discarding the supernatant, the cells were resuspended in 2.5 mL of S1 solution provided by the Frozen-EZ Yeast Transformation II kit (Zymo Research, USA). The suspension was centrifuged again at 2000 rpm for 10 min. The cell pellet was resuspended in 500 μL of S2 solution and aliquoted into sterilized Eppendorf tubes at 100 μL per tube. To each tube, 6 μL of DNA and 500 μL of S3 solution were added. The mixtures were incubated at 30 °C for 30 min, followed by a heat shock at 37 °C for 10 min. Subsequently, 2.5 mL of YPD medium was added to a 15 mL culture tube, and the contents of each Eppendorf tube were transferred to the culture tube and incubated at 30 °C for 12 h. The cultures were centrifuged at 2000 rpm for 10 min, the pellets were resuspended in 100 μL of sterilized deionized water, and the suspensions were spread onto appropriate selection plates.

##### 2.2.1.4 Colony PCR

Colony candidates were picked into PCR tubes using autoclaved toothpicks. To each PCR tube, 20 μL of 0.02 M sodium hydroxide (Fisher Chemical, USA) was added and mixed well by vortexing. The tubes were then heated at 98 °C for 10 min on a C1000 thermocycler (Bio-Rad, USA). Following heating, the PCR tubes were centrifuged for 3 min at 10,000 rpm. For the PCR reaction, 4 μL of the supernatant was used as a template, mixed with 0.5 μL of each primer and 5 μL of Phire Plant Direct PCR Master Mix (Thermo Fisher Scientific, USA). The PCR was set up on a thermocycler following the program recommended by the Phire Plant Direct PCR Master Mix protocol.

#### 2.2.2 New Strains Derived from Y8412

##### 2.2.2.1 *FAA1* and *GPD1* Random Insertion in Y8412 to Generate Y8412F^+^ and Y8412G^+^

The target gene expression cassettes for *FAA1* and *GPD1* were ligated with the selection marker expression cassette either by overlap extension PCR or by constructing a plasmid and then linearizing it (Figure S2). The combined target gene and selection marker expression cassette were then transformed into Y8412 using the Frozen-EZ Yeast Transformation II Kit (Zymo Research, USA). The transformants were grown on YPD hygromycin plates at 30 °C for 2 days. The resulting single colonies (10–20 colonies for each transformed plate) were picked for DNA purification and further confirmation by PCR to identify correct gene insertions, resulting in two new strains, Y8412F^+^ and Y8412G^+^.

##### 2.2.2.2 *TGL3* and *TGL4* Deletions in Y8412 to Generate Y8412T3^–^ and Y8412T4^–^

*TGL3* (YALI0D17534g) and *TGL4* (YALI0F10010g) genes were amplified from the ATCC20362 strain, and knockout cassettes were assembled using overlap extension PCR. The knockout cassette consisted of two fragments (Figure S1a). The upstream fragment contained a 1.5 kb sequence upstream of the *TGL3*/*TGL4* gene to be deleted and a partial *Hyg*^*R*^ expression cassette (Figure S2b). The downstream fragment contained a 1.5 kb sequence downstream of the *TGL3*/*TGL4* gene and the remaining portion of the *Hyg*^*R*^ expression cassette. The *Hyg*^*R*^ expression cassettes on the two fragments had an overlapping region of approximately 500 bp. These two fragments were transformed into Y8412. The new transformants were grown on YPD hygromycin plates at 30 °C for 2 days. The resulting single colonies (10–20 colonies for each transformed plate) were picked for DNA purification and further confirmation by PCR to identify correct gene insertions, resulting in the new strains Y8412T3^–^ and Y8412T4^–^. Since any single fragment inserted randomly via NHEJ would not express *Hyg*^*R*^, only Y8412 cells in which homologous recombination occurred between both upstream, downstream, and *Hyg*^*R*^ sequences (thereby reconstructing the complete *Hyg*^*R*^) would express hygromycin resistance. This approach allows for the screening of random insertions caused by NHEJ and avoids the need to disrupt KU70/KU80 genes, which can affect cell viability.

##### 2.2.2.3 *URA3* Knockout in Y8412T4^–^ to Generate Y8412T4^–^U^–^

The *URA3* gene was knocked out by homologous recombination methods (Figure S1b). A 25 bp sequence within the *URA3* gene was deleted using a linear cassette assembled by overlap extension PCR. The upstream fragment of the *URA3* gene was amplified from 554 bp before 5′ of the *URA3* ORF to 724 bp after the 5′ of the ORF, and the downstream fragment was amplified from 749 bp after the 5′ of the *URA3* ORF to 1113 bp after the 3′ of the *URA3* ORF from ATCC 20362 by PCR. The reverse primer for the upstream fragment and the forward primer for the downstream fragment contained extra sequences to ensure a 32 bp overlapping region between the 3′ of the upstream fragment and the 5′ of the downstream fragment. These two fragments were then used as templates and primers for assembly. The product of this step contained the *URA3* gene with a 25 bp deletion at 724 bp from the start of the *URA3* ORF. This product was then used as a template to amplify the *URA3* gene with the 25 bp deletion. The *URA3* gene with the 25 bp deletion was transformed into the Y8412T4^–^ strain. After homologous recombination between the exogenous *URA3* gene fragment and the chromosomal *URA3* gene in Y8412T4^–^, the *URA3* gene on the chromosome had the 25 bp deletion. Correct colonies were selected by their ability to grow on plates containing 0.2 g/L 5-fluoroorotic acid (5-FOA) and their inability to grow on yeast nitrogen base (YNB) plates without uracil.

##### 2.2.2.4 *TGL3* Knockout in Y8412T4^–^U^–^ to Generate Y8412T3^–^T4^–^

A linear *TGL3* knockout cassette with a *Ura3* selection marker was constructed by overlap extension PCR (Figure S2b). Then it was transformed into Y8412T4^–^U^–^. The transformants were grown on YNB uracil drop-out plates at 30 °C for 2–3 days. The resulting single colonies (10–20 colonies for each transformed plate) were picked for DNA purification and further confirmation by PCR to identify correct gene insertions, resulting in the new strain Y8412T3^–^T4^–^.

##### 2.2.2.5 *FAA1* and *GPD1* Random Insertion in Y8412T4^–^U^–^ to Generate Y8412F^+^T4^–^ and Y8412G^+^T4^–^

Linear *FAA1*/*GPD1* random insertion cassette with *Ura3* selection marker were constructed with overlap extension PCR (Figure S2c). Then, they were transformed into Y8412T4^–^U^–^. The transformants were grown on YNB uracil drop-out plates at 30 °C for 2–3 days. The resulting single colonies (10–20 colonies for each transformed plate) were picked for DNA purification and further confirmation by PCR to identify correct gene insertions, resulting in strains Y8412F^+^T4^–^ and Y8412G^+^T4^–^.

### 2.3 Hydrolyzing Waste Cooking Oil

A 1.0 L mixture containing 0.5 L of a 2× lipase solution and 0.5 L of WCO was prepared in a 1.5 L Biostat B-DCU fermentation system (Sartorius, Germany). The fermentor was maintained at 37 °C and agitated at 600 rpm. After 2 days of enzymatic reaction, the agitation was stopped for 15 min, allowing the mixture to separate into two distinct layers. Then the lower layer was removed and another 0.5 L of fresh 2× lipase solution was added. The hydrolysis process was continued under the same conditions for an additional 2 days.

### 2.4 Fed-Batch Flask Culture

Colonies with correct genome type confirmed by colony PCR were picked from appropriate selection plates using an inoculation loop and inoculated into 15 mL sterilized single-use culture tubes containing 2 mL YPD medium. After overnight cultivation, when the OD_600_ of each strain reached approximately 2.5, the cultures were centrifuged at 2000 rpm for 5 min, the supernatant was removed, and the cell pellet was resuspended with an appropriate volume of DI water to adjust the OD_600_ to 2.0. The fed-batch flask culture working medium was prepared by combining 2 mL of 5× fed-batch flask culture stock medium, 7 mL of water, and 1 mL of the standardized seed culture (OD_600_ = 2.0) to start the fed-batch flask culture at 30 °C, 250 rpm in an Innova-4000 incubator shaker.

Measurements of OD_600_ and residual glucose concentration were taken at 0, 48, 96, and 144 h. Tween 80 (10 μL) was added at 36 h. Hydrolyzed waste cooking oil (HWCO) was fed to each flask according to the following schedule: 44 μL at 36 h, and 22 μL at 48 and 72 h, respectively. Glucose was added at 48 and 96 h based on the residual glucose levels to maintain the residual glucose concentration at 40 g/L at these time points. At 144 h, 5 mL of broth was collected for DCW analysis, and a 500 μL sample was collected for lipid analysis.

### 2.5 Analysis of Flask Fermentation Samples

#### 2.5.1 Cell Density and Biomolecule Quantification

Cell density was determined by measuring the optical density at 600 nm (OD_600_) using a Genesys 10 Vis spectrophotometer (Thermo Fisher Scientific, USA). Glucose concentrations were measured using a YSI 2950 biochemical analyzer (YSI Incorporated, USA). DNA and RNA concentrations were quantified using a NanoDrop spectrophotometer (Thermo Fisher Scientific, USA).

#### 2.5.2 Dry Cell Weight Measurement

To determine the dry cell weight (DCW), 5 mL of the fed-batch flask broth was collected into a 15 mL centrifuge tube, and deionized (DI) water was added to reach a final volume of 15 mL. The mixture was centrifuged at 3000 rpm for 10 min, and the supernatant was carefully discarded to avoid losing any cells. The cell pellet was resuspended in 15 mL of DI water and centrifuged again. This washing step was repeated once more.

Empty aluminum trays were preweighed using an analytical balance, labeled accordingly, and the weights were recorded. The washed cell pellets were resuspended and transferred onto the corresponding aluminum trays. All trays were placed in a 65 °C incubator, and the weight of each tray was measured again after 2 days to determine the DCW.

#### 2.5.3 Lipids Analysis

Lipid analysis involved the transmethylation of lipids to fatty acid methyl esters (FAMEs), followed by analysis using an Agilent 8860 gas chromatograph equipped with a flame ionization detector (GC-FID) (Agilent, USA).

##### 2.5.3.1 Basic Transmethylation Methods for Analysis of Intracellular TAG Lipid

Under basic conditions, only TAGs react with methanol to form FAMEs. Therefore, this method was used to analyze the intracellular TAG concentration. Since there is no evidence that intracellular TAGs can transfer outside the cell membrane, only intracellular TAGs were analyzed.

First, 250 μL of broth was placed into a 2 mL screw-cap centrifuge tube and mixed with 125 μL of methanol, then centrifuged for 2 min at 10,000 rpm. The supernatant was transferred to a fresh 2 mL centrifuge tube for extracellular FFA analysis later. The cell pellet was resuspended with 2% sodium methoxide prepared by diluting 30% sodium methoxide (Thermo Fisher Scientific, USA), then approximately 100 μL of 0.5 mm glass beads and 20 μL of 5 g/L C15:0 triglyceride standard (Nu-Chek Prep, USA) in toluene were added. The mixture was vortexed for 30 min to fully homogenize the yeast cells and then shaken at 250 rpm for an additional 15 min. Subsequently, 200 μL of 1 M sodium chloride and 980 μL of heptane were added to each tube and vortexed for 2 min. A 400 μL aliquot of the supernatant was transferred into GC sample vials for analysis.

##### 2.5.3.2 Selective Acid Transmethylation Methods for Analysis of Intracellular and Extracellular FFAs

Selective acid transmethylation was employed to analyze FFAs content in lipid samples. This method utilizes the fact that only FFAs react with methanol under relatively weak acidic conditions at 50 °C, allowing for the transmethylation of both intracellular and extracellular FFAs.

Analysis of Intracellular FFAs

A 250 μL broth was placed in a 2 mL screw-cap centrifuge tube, and 125 μL of methanol was added. The mixture was centrifuged for 2 min at 10,000 rpm. The supernatant was transferred to the 2 mL centrifuge tube containing the supernatant from the intracellular TAG analysis. The cell pellet was resuspended with 1 mL of 0.9% hydrochloric acid (prepared by diluting 37% hydrochloric acid with methanol), approximately 100 μL of glass beads, and 20 μL of 5 g/L C17:0 FFA standard in toluene was added. The mixture was vortexed for 30 min. This mixture was then transferred to a screw-cap glass tube, and an additional 1 mL of 0.9% hydrochloric acid was used to wash the 2 mL centrifuge tube and transferred to the glass tube. The glass tube was incubated in a 50 °C water bath shaker and shaken for 10 min. Subsequently, 200 μL of 1 M sodium chloride and 980 μL of heptane were added to each glass tube, and the mixture was vortexed for 2 min. A 400 μL aliquot of the top layer was transferred to GC sample vials for analysis.

Analysis of Extracellular FFAs

The supernatants collected from the intracellular TAGs and FFAs transmethylation steps were combined in a 2 mL centrifuge tube, mixed with 250 μL of toluene and 225 μL of 1 M sodium chloride, and vortexed for 2 min. A 100 μL aliquot of the top layer was transferred to a screw-cap glass tube, to which 20 μL of 5 g/L C17:0 FFA standard and 2 mL of 0.9% hydrochloric acid were added. The mixture was incubated at 50 °C for 10 min. Subsequently, 200 μL of 1 M sodium chloride and 880 μL of heptane were added, and the mixture was vortexed for 2 min. A 400 μL aliquot of the top layer was transferred to GC sample vials.

##### 2.5.3.3 Liquid–Gas-FID Chromatography

The GC system used a GC-grade helium (Airgas, USA) as the carrier gas, a hydrogen generator (Peak Scientific, USA) to produce hydrogen as fuel gas, and zero-grade air (Airgas, USA) to provide oxygen for maintaining the flame in the FID detector. The column used for FAMEs analysis was DB-fastFAME (Agilent, USA).

The inlet temperature was set at 250 °C, and the inlet pressure was maintained at 35 psi. The oven temperature program started at 80 °C and was held for 1 min, then increased at a rate of 35 °C per minute to 194 °C, where it was held for 1 min. Subsequently, the temperature increased at 5 °C per minute to 240 °C and was held for 0.5 min. The FID detector was set at 290 °C, with an airflow of 400 mL/min, hydrogen flow of 30 mL/min, and makeup flow of 25 mL/min.

Standards of each fatty acid in methyl ester form, including PA (C16:0), PLA (C16:1), SA (C18:0), OA (C18:1), LA (C18:2), ALA (C18:3), EDA (C20:2), DGLA (C20:3), ERA (C20:3), ARA (C20:4), ETA (C20:4), and EPA (C20:5), were purchased from Nu-Chek Prep. To generate standard curves for each fatty acid, seven standard samples were prepared, containing 25, 30, 40, 55, 75, 100, and 130 mg/L of each respective fatty acid methyl ester. All standard samples also contained 50 mg/L each of C15:0 and C17:0 fatty acid methyl esters as internal standards.

Based on the retention times of these standards, each chromatographic peak was identified according to standard fatty acid profile. The peak area data for each single fatty acid (SA) and the peak area for the internal standard (C15:0 or C17:0) (CA) were then collected. The following formula was used to calculate the concentration of each fatty acid
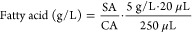


### 2.6 RT-qPCR

Fed-batch flask cultures of the Y8412 strain were grown with glucose only and cofed with glucose and HWCO, and samples were collected at 20, 72, and 120 h. At each time point, two samples were taken from each flask: one for measuring the expression level of the housekeeping gene *GAPDH* and one for the target gene *TGL4*. Total RNA was extracted from each sample following the protocol of the YeaStar RNA kit (Zymo Research, USA). The RNA concentration was measured using a NanoDrop spectrophotometer (Thermo Fisher Scientific, USA). RT-qPCR was performed using the Luna Universal One-Step RT-qPCR Kit (New England Biolabs, USA) on a CFX96 Real-Time PCR Detection System (Bio-Rad, USA). The quantification cycle (Cq) values of the target gene experimental (TE), the housekeeping gene experimental (HE), the target gene control, and the housekeeping gene control were collected, and data were analyzed using the ΔΔCq method in Excel.
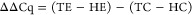

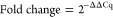


## 3 Results and Discussion

### 3.1 EPA Production from *Y. lipolytica* Y8412 with Co-Feeding of Glucose and HWCO

Our previous data in the wax ester biosynthesis project indicated that feeding*Y. lipolytica* with hydrolyzed waste cooking oil (HWCO) enhanced production titer and yield compared to using WCO directly.^35^ It seemed the activity of lipases produced by*Y. lipolytica* itself may become limited in the middle of the fermentation, which caused slow uptake of WCO as the original WCO contained about 14% FFAs and 86% TAGs (Figure S7). Therefore, we prehydrolyzed all WCO before conducting cofeeding studies with glucose in flask fermentation experiments. After being hydrolyzed, it contained 82% FFAs and 18% TAGs (Figure S7), which benefited the oil uptake in the form of FFAs in the stationary phase of the fermentation.

**Figure 2:**
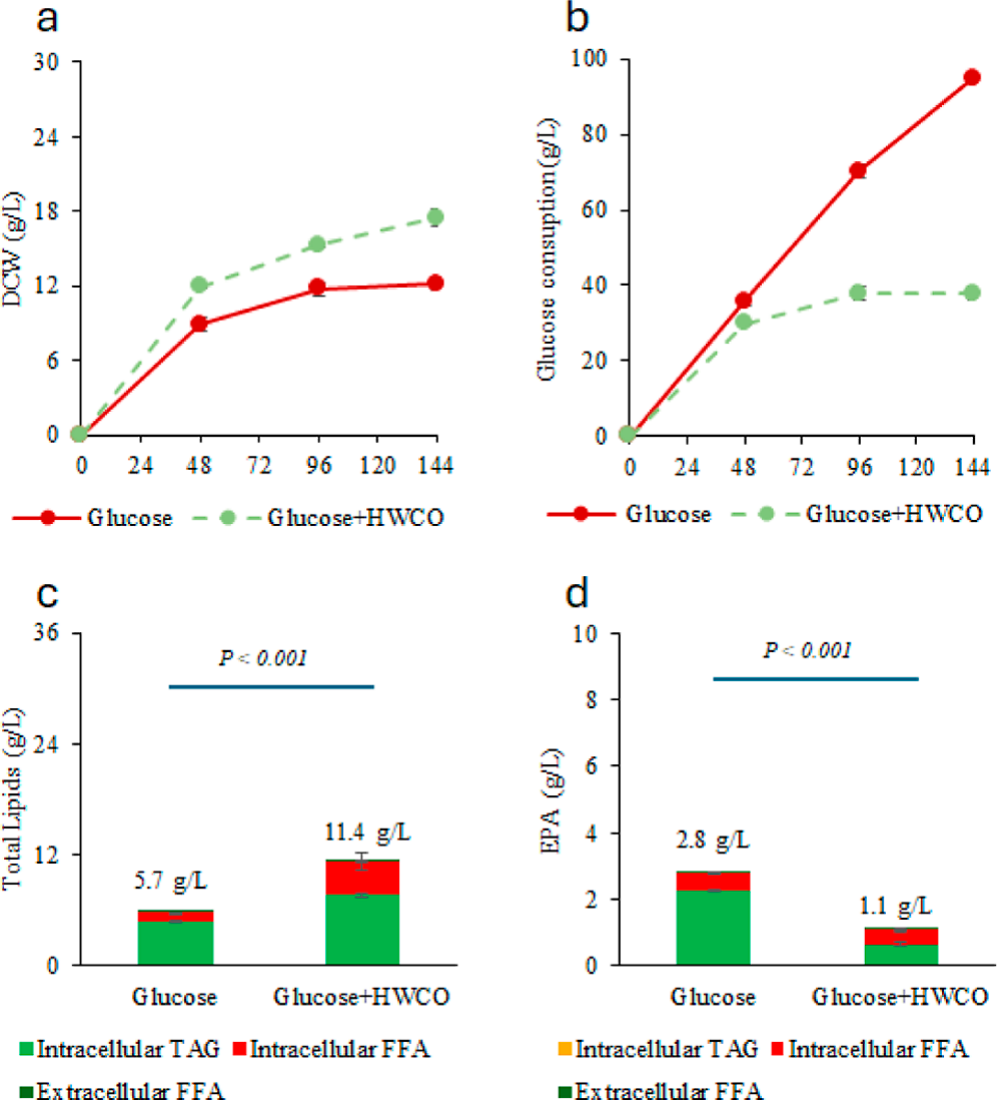
Comparison between the fed-batch flask cultures of *Y. lipolytica* Y8412 with glucose-only feeding and cofeeding of glucose and HWCO. (a) Cell density, two biological replicates (b) total glucose consumption, (c) total lipid production, (d) total EPA production. Cells were cultured in duplicate flasks. At each indicated time point, a single sample was collected from each flask and analyzed in duplicate for lipids. Data points represent the average values of these technical replicates, and error bars indicate the standard deviation.

The fed-batch flask culture results for strain Y8412 using glucose only and cofeeding with glucose and HWCO are shown in Figure 2. The profile of individual fatty acids from the produced lipids and the original WCO and HWCO are also shown in Figures S3–S6. After cofeeding an extra 7.9 g/L of HWCO to the Y8412 strain, the DCW (Figure 2a) at 144 h was 45% higher compared to strain Y8412 fed with glucose only. However, the run with cofeeding of HWCO consumed only 37.9 g/L of glucose in total, significantly less than the run fed with glucose only, 94.8 g/L (Figure 2b). It seemed that HWCO partially replaced glucose as the carbon source.

The*Y. lipolytica*Y8412 strain is deficient in β-oxidation due to the *pex3* knockout and while lipids cannot be metabolized to acetyl-CoA to provide energy for the cells,^24^ they can still be internalized and stored as lipid droplets (LD). At 144 h, the total lipids increased from 5.7 to 11.4 g/L from the cofeeding (Figure 2c). However, the increase in total lipid did not lead to an increase in EPA. The total EPA actually decreased from 2.8 to 1.1 g/L after cofeeding with HWCO (Figure 2d). Further investigation into the increase in total fatty acids revealed that the concentration of FFAs increased from 0.9 to 3.7 g/L, a 310% increase. High levels of FFAs are toxic to cells, thereby decreasing cell viability and likely lowering the efficiency of all enzymes involved in EPA synthesis.

Therefore, we aimed to reduce intracellular FFA levels by converting them into TAGs, which should help maintain cell viability and enhance the effectiveness of EPA synthesis. Moreover, EPA produced in TAG form not only has higher bioavailability for humans but also avoids the need of re-esterifying FFAs into TAGs during the downstream processing, making the product more valuable. It was our belief that re-engineering Y8412 to convert these excess FFAs is crucial in converting WCO to EPA.

### 3.2 Overexpression of *FAA1* and *GPD1* to Convert Intracellular FFAs into TAGs

**Figure 3:**
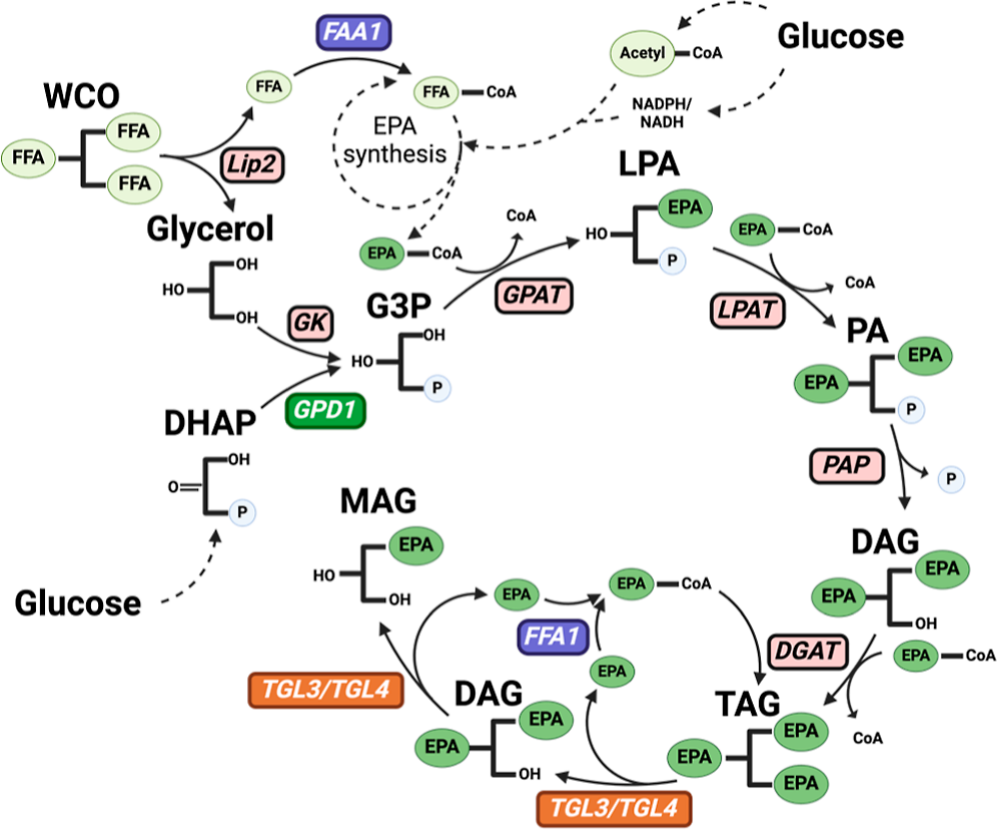
An overview of TAG synthesis pathway. Metabolite abbreviations: dihydroxyacetone phosphate (DHAP), glycerol-3-phosphate (G3P), lysophosphatidic acid (LPA), phosphatidic acid (PA), diacylglycerol (DAG), monoacylglycerol (MAG).

The structure of TAGs consists of a glycerol backbone with three FFAs. Figure 3 shows that the TAG synthetic pathway starts by binding activated FFAs (FFA-CoA) one by one to the glycerol-3-phosphate (G3P) backbone.

*FAA1* activates FFAs by adding a CoA group, forming FFA-CoA, while *GPD1* converts dihydroxyacetone phosphate into glycerol-3-phosphate (G3P). Overexpressing *FAA1* or *GPD1* in the Y8412 strain was expected to increase FFA-CoA and G3P, enhancing TAG synthesis and thereby reducing FFA toxicity and boosting EPA production as TAG.

**Figure 4:**
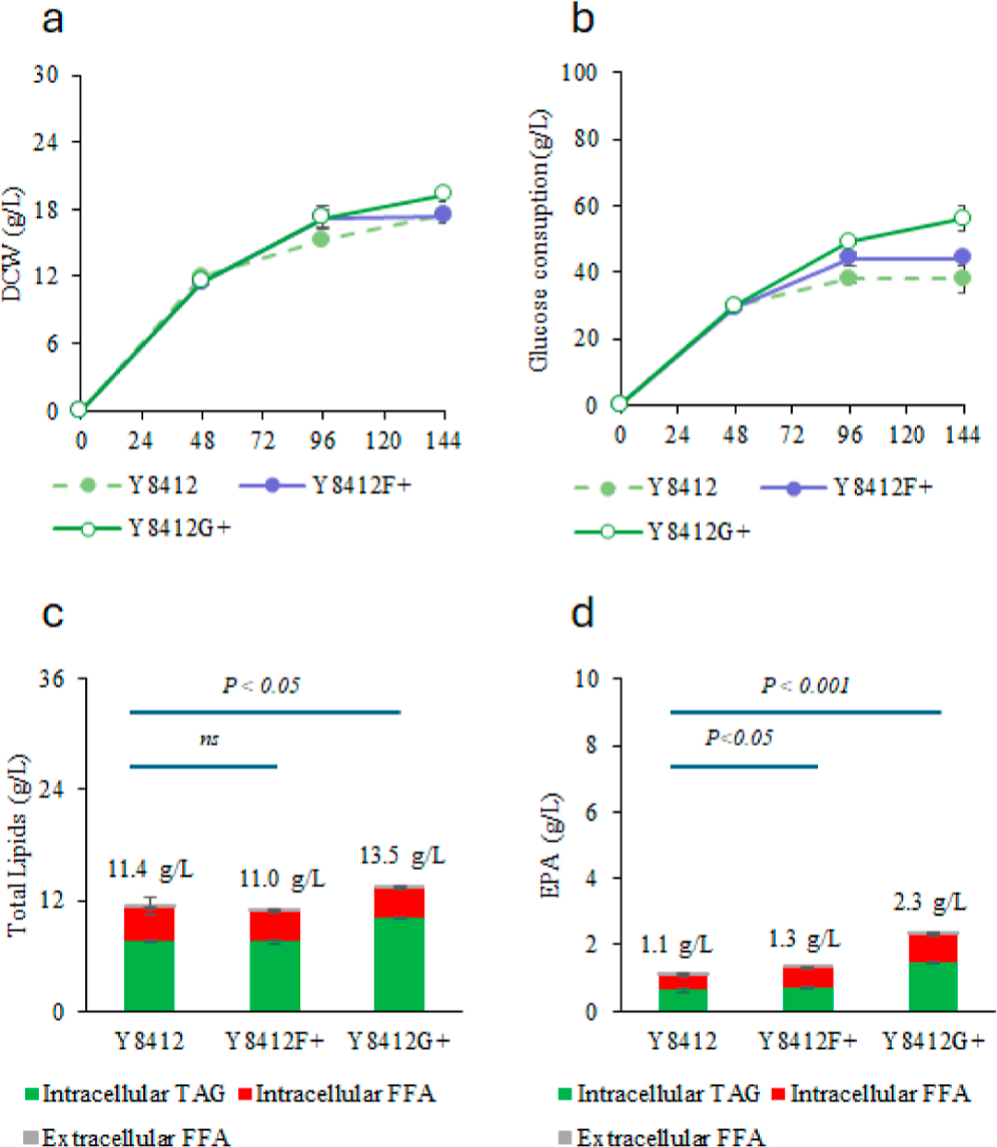
Comparison of fed-batch flask cultures of *Y. lipolytica* Y8412, Y8412F^+^, and Y8412G^+^ strains with cofeeding of glucose and HWCO. (a) Cell density, (b) total glucose consumption, (c) total lipid production, (d) total EPA production. Cells were cultured in duplicate flasks. At each indicated time point, a single sample was collected from each flask and analyzed in duplicate for lipids. Data points represent the average values of these technical replicates, and error bars indicate the standard deviation.

The results of the fed-batch flask cultures with cofeeding of glucose and HWCO (Figure 4a) show that the cell densities of the two re-engineered strains Y8412F^+^ and Y8412G^+^ with overexpression of *FAA1* and *GPD1*, respectively, are significantly higher than that of the parent strain Y8412 in a similar condition. While strain Y8412 consumed only 37.9 g/L of glucose, Y8412F^+^ and Y8412G^+^ consumed 44.0 and 56.2 g/L of glucose, respectively (Figure 4b). Y8412F^+^ accumulated 11.0 g/L of total lipids, which was similar to that of Y8412 (Figure 4c). However, the FFAs produced by Y8412F^+^ were 3.35 g/L, slightly lower than that of Y8412 (3.72 g/L), and the EPA titer was 1.3 g/L, which was 22% higher than that of Y8412. Y8412G^+^ produced 13.5 g/L of total lipid, which was 19% more than that of Y8412, and 3.2 g/L of FFAs, which was 14% less than the parent strain. Most importantly, Y8412G^+^ produces 2.3 g/L of EPA (Figure 4d), which was 115% higher than that of Y8412. These results suggest that the *FAA1* gene may not be the primary limiting step in converting FFAs to TAGs. However, the increased EPA titer indicates that *FAA1* may help activate more fatty acids from WCO to fatty acyl-CoA, allowing greater utilization of WCO for further elongation and desaturation in EPA synthesis. The Y8412G^+^ strain shows higher viability than Y8412, thus producing more EPA. Overexpressing *GPD1* further facilitates the conversion of FFAs to TAGs. However, the EPA produced by Y8412F^+^ and Y8412G^+^ still had a significant portion of FFA as compared to the parent strain Y8412. Though the total EPA produced by Y8412G^+^ (2.3 g/L) was higher than Y8412 (1.1 g/L) when the flask culture was cofed with HWCO and glucose, it was still less than Y8412 fed with glucose only (2.8 g/L). More effort should be made to further reduce the formation of FFAs so that significantly higher EPA production can be achieved.

### 3.3 Deletion of *TGL3*/*4* Genes for Enhanced Lipid and EPA Production

**Figure 5:**
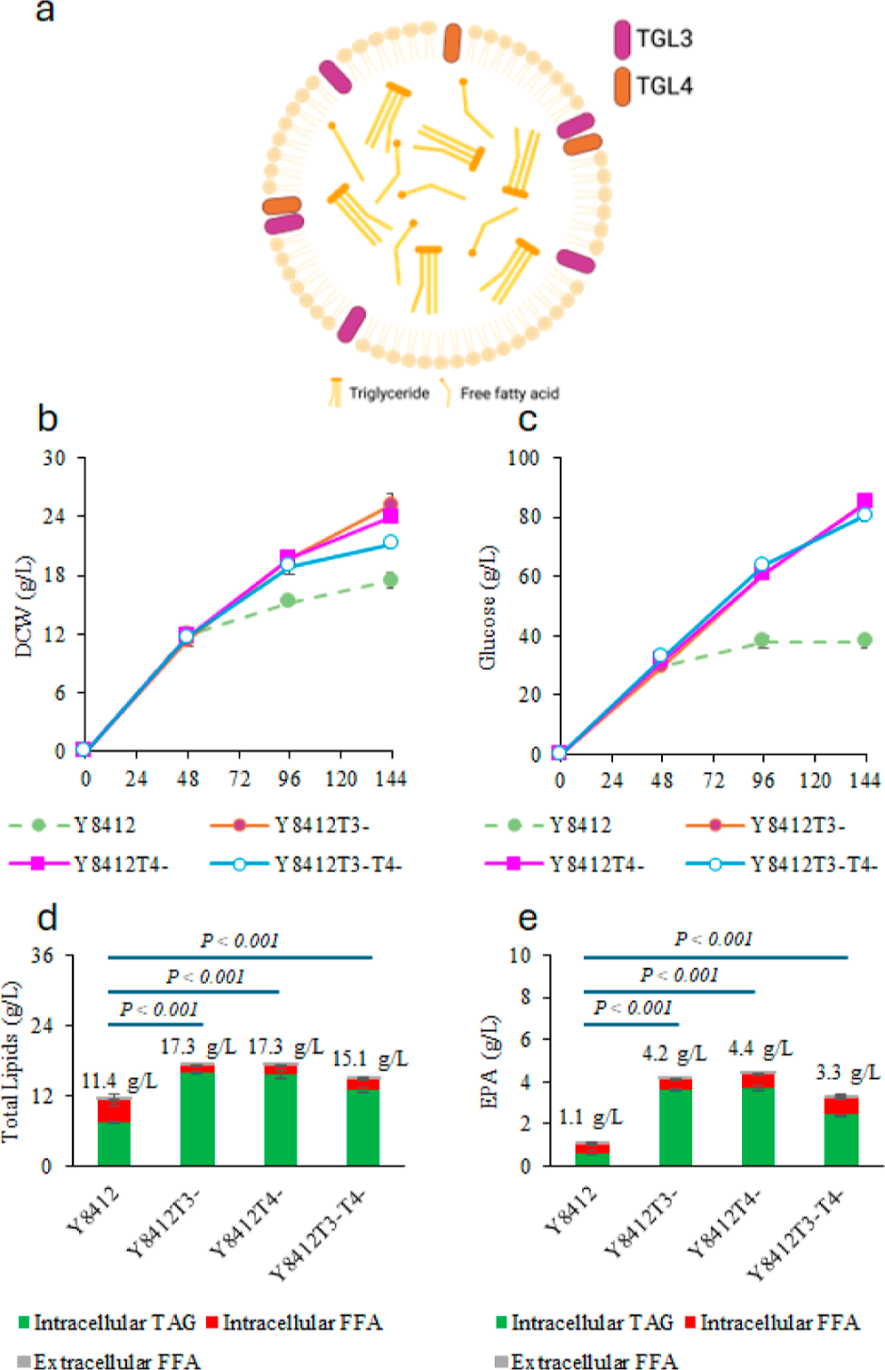
Deleting the *TGL3*/4 gene in strain Y8412 for enhanced EPA production from glucose and HWCO. (a) A schematic diagram of intracellular lipases *TGL3* and *TGL4*. (b–e) Comparison between fed-batch cultures of Y8412, Y8412T3^–^, Y8412T4^–^, and Y8412T3^–^T4^–^ strains cofed with glucose and HWCO. (b) Cell density, (c) Total glucose consumption, (d) Total lipid production, (e) Total EPA production. Cells were cultured in duplicate flasks. At each indicated time point, a single sample was collected from each flask and analyzed in duplicate for lipids. Data points represent the average values of these technical replicates, and error bars indicate the standard deviation.

*TGL3* and *TGL4* are two major intracellular lipases integrated into LD (Figure 5a). When yeast cells need to utilize the TAGs stored in LDs, *TGL3* and *TGL4* degrade TAGs into FFAs (Figure 3). The FFAs can then be further degraded into acetyl-CoA via β-oxidation to provide carbon sources for energy production or the synthesis of building blocks for other cellular activities. However, Y8412 is a *Pex3* mutant strain lacking the β-oxidation pathway inside the cells, leading to the accumulation of high levels of FFAs, which may cause toxicity to the yeast cells and lead to decreased cell density and lower cellular activities required for omega-3 fatty acid synthesis.

First, we investigated how intracellular lipid levels affected the changes in TGL expression in strain Y8412 during the fed-batch flask culture process. As shown in Table 2, the transcription level of *TGL4* increases by 1.7-fold from 20 to 72 h when Y8412 was fed with glucose only. However, the *TGL4* expression level increased by 3.77-fold from 20 to 72 h when the strain was cofed with glucose and HWCO. These results suggest that *TGL4* overexpression increased under HWCO cofeeding conditions likely leading to the higher FFA accumulation in strain Y8412. Therefore, it is expected that deleting the *TGL3*/*4* gene may help keep the lipid in TAG form and reduce the intracellular FFAs. Thus, *TGL3* and *TGL4* were deleted in Y8412 to generate strains Y8412T3^–^ and Y8412T4^–^, respectively. A *TGL3* and *TGL4* double knockout strain Y8412T3^–^T4^–^ was also generated by deleting *TGL3* in strain Y8412T4^–^U^–^ strain (Figure S2b).

**Table 2:** Fold-Increase of *TGL4* Expression Levels (Measured by RT-PCR) from 20 to 72 h for Strain Y8412 in Fed-Batch Flask Cultures

carbon source	fold-increase of *TGL4* expression (72 h vs 20 h)
glucose	1.70
glucose & HWCO	3.77

The fed-batch flask culture results of these strains show that the cell densities of Y8412T3^–^, Y8412T4^–^, and Y8412T3^–^T4^–^ were 44, 30, and 15% higher than that of strain Y8412, respectively (Figure 5b). At the same time, their glucose consumption was also 124, 124, and 113% higher than that of Y8412, respectively (Figure 5c). In addition, the total lipid production of Y8412T3^–^, Y8412T4^–^, and Y8412T3^–^T4^–^ were 52, 52, and 32% higher than Y8412, respectively (Figure 5d). The microscope picture also observes larger lipid droplets (Figure S8). Further analysis of the FFA levels in Y8412T3^–^, Y8412T4^–^, and Y8412T3^–^T4^–^ showed that their FFA concentrations were 63, 60, and 49% lower than in the parent strain Y8412, respectively (Figure 5d). The significant decrease in FFA levels indicates that *TGL3* and *TGL4* gene expression was the major reason for FFA accumulation. At lower FFA levels, the newly engineered cells accumulated more biomass and consumed more glucose, indicating that the cells were healthier and more active in EPA biosynthesis. This confirmed our initial belief that intracellular lipase (*TGL3*/*4*) overexpression occurs when a high level of lipid accumulates to produce high levels of FFAs, which are toxic to the yeast cells and lead to lower cell density and glucose consumption rates. However, a double knockout of TGL3 and 4 did not further improve EPA. It seems maintaining a certain level of FFAs is important for both EPA synthesis and cells’ viability.

The EPA titers in TAG form for Y8412T3^–^, Y8412T4^–^, and Y8412T3^–^T4^–^ were 467, 280, and 282% higher than that of Y8412, respectively, and their total EPA titers in both TAG and FFA forms also increased by 281, 301, and 207%, respectively (Figure 5e, Table 3). As compared with strain Y8412 fed with glucose only, the total EPA titers (in both TAG and FFA forms) produced from Y8412T3^–^, Y8412T4^–^, and Y8412T3^–^T4^–^ with cofeeding of glucose and HWCO increased by 49, 57, and 20%, respectively. It seemed that a single deletion of *TGL3* or *TGL4* led to higher lipid and EPA production than the double deletions of both genes, with the *TGL4* deletion slightly better than the *TGL3* deletion. The results also indicated that with the newly engineered strain Y8412T4^–^, using only a small amount of WCO (7.6 g/L) to partially replace 10.5% of the total glucose consumed (94.8 g/L) led to a 57% increase in the overall EPA titer. It suggests that the increased lipid accumulation caused by cofeeding HWCO significantly contributes to additional EPA synthesis.

**Table 3:** Summary of Total Lipid and EPA Production from Glucose or Glucose + HWCO by the Metabolically Engineered *Y. lipolytica* Strains in Fed-Batch Flask Culture

strain	Y8412		Y8412F^+^	Y8412G^+^	Y8412T3^–^	Y8412T4^–^	Y8412T3^–^T4^–^	Y8412F^+^T4^–^	Y8412G^+^T4^–^
carbon source	glucose	glucose + HWCO	glucose + HWCO	glucose + HWCO	glucose + HWCO	glucose + HWCO	glucose + HWCO	glucose + HWCO	glucose + HWCO
EPA as TAG (g/L)	2.3 ± 0.04	0.6 ± 0.06	0.7 ± 0.02	1.5 ± 0.04	3.6 ± 0.05	3.7 ± 0.12	2.4 ± 0.03	3.1 ± 0.04	2.7 ± 0.16
EPA as FFA (g/L)	0.5 ± 0.03	0.5 ± 0.06	0.6 ± 0.03	0.9 ± 0.03	0.5 ± 0.04	0.7 ± 0.03	0.9 ± 0.04	0.7 ± 0.01	1.1 ± 0.01
total EPA (TAG + FFA) (g/L)	2.8 ± 0.07	1.1 ± 0.11	1.3 ± 0.02	2.3 ± 0.07	4.2 ± 0.02	4.4 ± 0.15	3.3 ± 0.02	3.8 ± 0.07	3.8 ± 0.17
total lipid as TAGs (g/L)	4.8 ± 0.03	7.6 ± 0.16	7.6 ± 0.18	10.2 ± 0.10	15.9 ± 0.08	15.7 ± 0.74	13.0 ± 0.34	15.1 ± 0.72	13.8 ± 0.03
total lipid as FFAs (g/L)	0.9 ± 0.06	3.8 ± 0.93	3.4 ± 0.12	3.3 ± 0.09	1.4 ± 0.05	1.5 ± 0.02	2.1 ± 0.11	1.5 ± 0.06	2.2 ± 0.11
total lipid (TAGs + FFAs) (g/L)	5.7 ± 0.06	11.4 ± 0.89	11.0 ± 0.06	13.5 ± 0.18	17.3 ± 0.05	17.3 ± 0.75	15.1 ± 0.24	16.7 ± 0.78	16 ± 0.10
EPA/total lipid	49%	9.6%	12.2%	17.4%	24%	25.4%	22.2%	22.8%	23.9%
TAGs/DCW	39.1%	43.3%	43.4%	52.8%	63.3%	65.8%	61.4%	70.7%	64.3%
EPA/DCW	19.1%	4.2%	5.3%	9.2%	15.2%	16.7%	13.6%	16.1%	15.4%
glucose consumed (g/L)	94.8 ± 0.0	37.9 ± 1.8	44.0 ± 2.1	56.2 ± 4.0	84.8 ± 0.0	84.8 ± 0.0	80.4 ± 2.1	82.5 ± 2.3	83.7 ± 1.3
HWCO fed (g/L)^a^	0.0	7.6	7.6	7.6	7.6	7.6	7.6	7.6	7.6
EPA Carbon yield – *Y*E_/_GO (% Mol/Mol)^b^	3.50%	2.48%	2.63%	3.85%	5.01%	5.24%	4.12%	4.64%	4.58%

a Based on lipid analysis of the end-of-run samples, all HWCO fed was consumed at the end of the experiments.

b EPA carbon yield *Y*_E/GO_ is defined by the carbon from produced EPA/carbon from initial glucose in medium and total HWCO fed during the fed-batch flask culture. The carbon from yeast extract and other nutrients was not included in the carbon yield calculation. It is calculated by the equation: *Y*_E/GO_ = [0.79*C*_EPA_/(0.4*C*_Glc_ + 0.77*C*_HWCO_)](*V*_f_/*V*_0_) × 100%, where *C*_EPA_, *C*_Glc_, and *C*_HWCO_ represent the total EPA titer, glucose consumed, and HWCO fed, respectively, based on initial working volume in flask culture. *V*_f_ and *V*_0_ stand for the final and initial culture volume. In this study, it was found *V*_f_/*V*_0_ ≈ 0.6 due to volume loss caused by evaporation. The numerical coefficients 0.79, 0.4, and 0.77 are the carbon content (g/g) in EPA, glucose, and HWCO, respectively.

### 3.4 Combined Effect of Overexpression of *FAA1* or *GPD1* and Deletion of *TGL4*

From the results shown earlier in Section 3.2, overexpressing *FAA1* and *GPD1* both increased conversion of FFAs into TAGs, and the data in Section 3.3 also showed that *TGL4* deletion prevented TAGs from being degraded into FFAs. Therefore, we next investigated whether combining *TGL4* deletion and overexpressing *FAA1* or *GPD1* was additive, resulting in two new strains Y8412F^+^T4^–^ and Y8412G^+^T4^–^.

**Figure 6:**
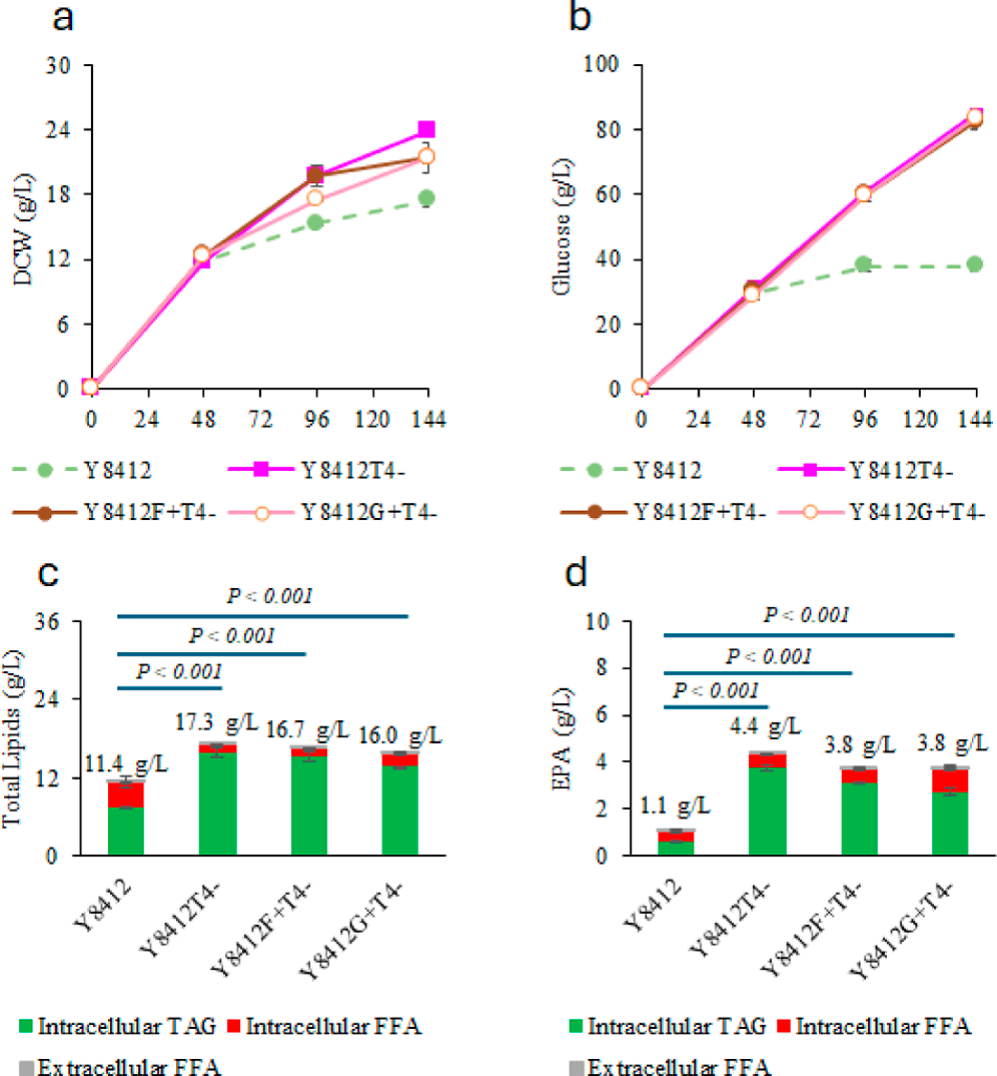
Comparison of the fed-batch flask culture of Y8412, Y8412T4^–^U^–^, Y8412F^+^T4^–^, and Y8412G^+^T4^–^ with cofeeding of glucose and HWCO. (a) Cell density, (b) total glucose consumption, (c) total lipid production, (d) total EPA production. Cells were cultured in duplicate flasks. At each indicated time point, a single sample was collected from each flask and analyzed in duplicate for lipids. Data points represent the average values of these technical replicates, and error bars indicate the standard deviation.

The fed-batch flask culture results with cofeeding of glucose and HWCO show that the cell densities of Y8412F^+^T4^–^ and Y8412G^+^T4^–^ were lower than those of Y8412T4^–^ (Figure 6a), but their total glucose consumption is similar (Figure 6b). Their total lipid titers (in both TAG and FFA forms) were slightly lower than that of Y8412T4^–^ though they were still significantly higher than that of Y8412 (Figure 6c). Moreover, the EPA titers of Y8412F^+^T4^–^ and Y8412G^+^T4^–^ were also nearly 15% lower than that of Y8412T4^–^ (Figure 6d). Though either overexpression of *FFA1*/*GPD1* or deletion of *TGL4* increased lipid and EPA production, it is unclear why a combination of both effects did not lead to additional improvements in either total lipid or EPA production. On the one hand, maintaining certain levels of FFAs or fatty acyl-CoAs is important for the biosynthesis of EPA as more elongation and desaturation steps are required in the pathway (Figure 1). On the other hand, converting the produced FFA-based EPA into its TAG form and storing it in lipid bodies is also necessary to keep cell viability and activity. This study thus uncovers future opportunities to optimize the overall fatty acid synthesis, omega-3 fatty synthesis, and lipid formation and remodeling.

Table 3 summarizes the lipid and EPA production from glucose only or glucose and HWCO by the original strain Y8412 and the newly engineered strains in this study during the fed-batch flask cultures. Y8412T4^–^ remains the best strain for the production of omega-3 EPA from glucose and HWCO. Interestingly, we observed the total EPA titer for Y8412T4^–^ fed with glucose and HWCO increased from 2.8 to 4.4 g/L when compared with the case of Y8412 fed with glucose only. This means that an additional 57% EPA production (1.6 g/L) was achieved by using only 7.6 g/L HWCO to replace 10 g/L of the total glucose consumed, which was only 10.5% of the total glucose consumed (94.8 g/L) by strain Y8412 fed with glucose. The overall carbon yield of EPA (mole carbon in EPA/mol carbon from both glucose and HWCO consumed) was also improved by nearly 50%.

Based on the fatty acid synthesis mechanism and metabolic pathway analysis,^36^ 6.5 mol glucose is required to provide acetyl-CoA and NADPH for synthesis of 1 mol EPA, leading to a theoretical mass yield for EPA from glucose *Y*_EPA/Glc_ = 0.26 g/g. If using fatty acid, e.g. oleic acid (C18:1, OLA), as the main carbon source, then 1 mol OLA and 2/3 mol glucose are required to synthesize 1 mol EPA, leading to a theoretical mass yield of EPA from OLA *Y*_EPA/OLA_ = 1.07 g/g and a theoretical carbon yield of EPA from both glucose OLA *Y*_EPA/GO_ = 0.91 mol/mol. Our recent study showed that the biomass yield was 0.25–0.31 g/g for glucose and 0.40–0.44 g/g for WCO, respectively.^20^ It seems that using WCO to partially replace glucose brings advantages in both EPA and biomass yield, which was consistent with our observations in this study.

Using WCO as substrate may lead to challenges in fermentation, especially limitations in mixing and mass transfer in bioreactor vessel due to the use of a hydrophobic substrate. In our recent studies, we have addressed issues by using pitched-blade impellers, increased agitation speed within certain aeration rate range through both experimental and CFD studies.^20,32,34^ Although mixing and foaming are not major issues in our shake flask fermentation studies as only 0.76% WCO was used in the media and no aeration was required, we expect further optimization of bioreactor design and operation is needed during the scale-up process.

The WCO used in this study was originally canola oil. GC analysis of the WCO shows that WCO contains 64% oleic acid (OA, C18:1), 17% linoleic acid (LA, C18:2), 8% palmitic acid (PA, C16:0), 5% alpha-linolenic acid (ALA, C18:3), and 4% stearic acid (C18:0), as shown in Figure S6. We believe that fatty acid composition in WCO is a key factor to consider for EPA synthesis. Higher content of LA, e.g. in soybean oil, may place less metabolic stress on the yeast as there would be a reduced need to desaturate OA into LA, thus requiring fewer cofactors for improved EPA production, which should be explored in future studies.

## 4 Conclusion

In this study, metabolic engineering strategies for enhanced omega-3 EPA production from HWCO were explored by using an engineered *Y. lipolytica* strain Y8412, originally developed for producing EPA from glucose. Co-feeding HWCO alongside glucose increased cell density and total lipid content in Y8412. However, the use of HWCO also led to the accumulation of intracellular FFAs, which are toxic to the yeast cells and reduce EPA synthesis. To address the FFA toxicity, *FAA1* and *GPD1* genes were overexpressed in Y8412 to convert excess FFAs into TAGs. The newly generated strains Y8412F^+^ and Y8412G^+^ exhibited an 11–13% decrease in FFA accumulation and an increase in EPA production by 18 and 110%, respectively, when HWCO was cofed with glucose. However, their total EPA titers remained lower than those achieved by Y8412 fed with glucose alone.

Additional effort was made to delete *TGL3* and *TGL4* in Y8412, which encode intracellular lipases to degrade TAGs into FFAs. The *TGL4* knockout strain Y8412T4^–^ exhibited a 61% decrease in intracellular FFAs and a 300% increase in EPA production over the parent strain Y8412 when both HWCO and glucose were fed as the carbon source. The produced EPA by Y8412T4^–^ fed with HWCO and glucose was also 57% higher than that by Y8412 fed with glucose only, showing that preventing the formed TAGs from being degraded into FFAs is more effective than merely enhancing TAG synthesis. The deletion of *TGL3* resulted in slightly lower EPA production than the deletion of *TGL4* in Y8412, but was still more efficient than the double deletion of *TGL3* and *TGL4*. Combining the overexpression of *FAA1* or *GPD1* with *TGL4* deletion did not provide additional advantages, indicating that eliminating TAG degradation via *TGL4* deletion sufficiently alleviated FFA toxicity.

To further enhance EPA production from HWCO, it is expected that a comprehensive engineering strategy and pathway design should be explored to optimize the overall fatty acid synthesis, omega-3 fatty synthesis, and lipid formation and remodeling in the yeast. Additional fermentation process development efforts should also be made for efficient uptake and conversion of the hydrophobic substrate in bioreactors. Our research work provides a new biomanufacturing route for sustainable and low-cost production of omega-3 fatty acids from waste resources.

## Supporting Information Available

General strategy for using homologous recombination to create *Hyg*^R^ selection maker or to delete the *Ura3* selection marker; Plasmids, linear DNA, and DNA fragments used in this study to generate new engineered strains from Y8412; Detailed fatty acid profiles determined by GC analysis for the lipid produced by Y8412 and newly engineered strains in fed-batch flask fermentation; Fatty acid profile of WCO and HWCO; Microscope pictures of engineered *Y. lipolytica* cells from the fed-batch flask cultures; All primers used in this study (PDF)

## Abbreviations

PUFAs: polyunsaturated fatty acids
EPA: eicosapentaenoic acid
WCO: waste cooking oil
FFA: free fatty acid
TAG: triglyceride
TAL: triacetic acid lactone
PA: palmitic acid
OA: oleic acid
LA: linoleic acid
EDA: eicosadienoic acid
DGLA: dihomo-γ-linolenic acid
ETA: eicosatetraenoic acid
Δ9D: Δ9 desaturase
Δ12D: Δ12 desaturase
Δ9E: Δ9 elongase
Δ8D: Δ8 desaturase
Δ5d: Δ5 desaturase
Δ17D: Δ17 desaturase
Δ15D: Δ15 desaturase
DCW: dry cell weight
HWCO: hydrolyzed waste cooking oil
HygR: hygromycin-resistance gene
NHEJ: nonhomologous end-joining
HR: homologous recombination
DHAP: dihydroxyacetone phosphate
G3P: glycerol-3-phosphate
LPA: lysophosphatidic acid
PA: phosphatidic acid
DAG: diacylglycerol
MAG: monoacylglycerol
LD: lipid droplet
FAMEs: fatty acid methyl esters
