# Supplementary material for: Metabolic Engineering of Yarrowia lipolytica for Conversion of Waste Cooking Oil into Omega-3 Eicosapentaenoic Acid
Source: ACS Eng Au. 2025 Feb 13;5(2):128–39. doi: 10.1021/acsengineeringau.4c00053 (PMC12006985; doi:10.1021/acsengineeringau.4c00053)
Supplement: Supplementary file 1 — eg4c00053_si_001.pdf [file eg4c00053_si_001.pdf]

Supplementary Information

Metabolic Engineering of *Yarrowia lipolytica* for Conversion of Waste Cooking Oil into Omega-3 Eicosapentaenoic Acid

Jiansong Qin [1], Na Liu [1], Umer Abid [1], Sarah M. Coleman [2], Yongdan Wang [1], Qiang Fu [1], Seongkyu Yoon [1], Hal S. Alper [2*], Dongming Xie [1*]

[1] Department of Chemical Engineering, University of Massachusetts Lowell, Lowell, MA 01854, USA.

[2] McKetta Department of Chemical Engineering, The University of Texas at Austin, Austin, TX 78712, USA.

*Correspondence:

Dr. Dongming Xie, Email: Dongming_Xie@uml.edu,

Dr. Hal S. Alper, Email: halper@che.utexas.edu

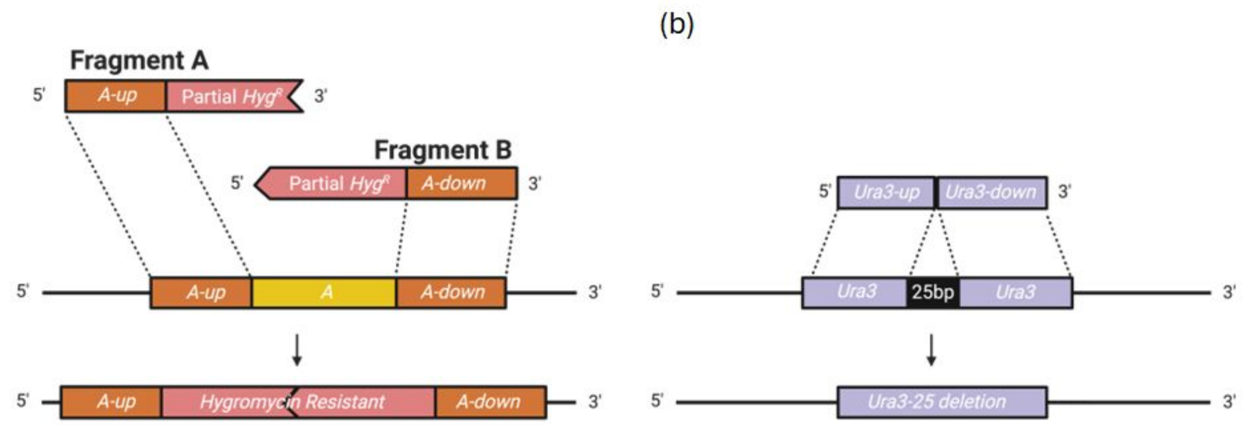

Figure S1. Using homologous recombination to create Hyg^R selection maker or to delete the *Ura3* selection marker. (a) Two linear cassettes for the split selection marker homologous recombination method. (b) Linear cassette for the *Ura3* homologous recombination knockout method.

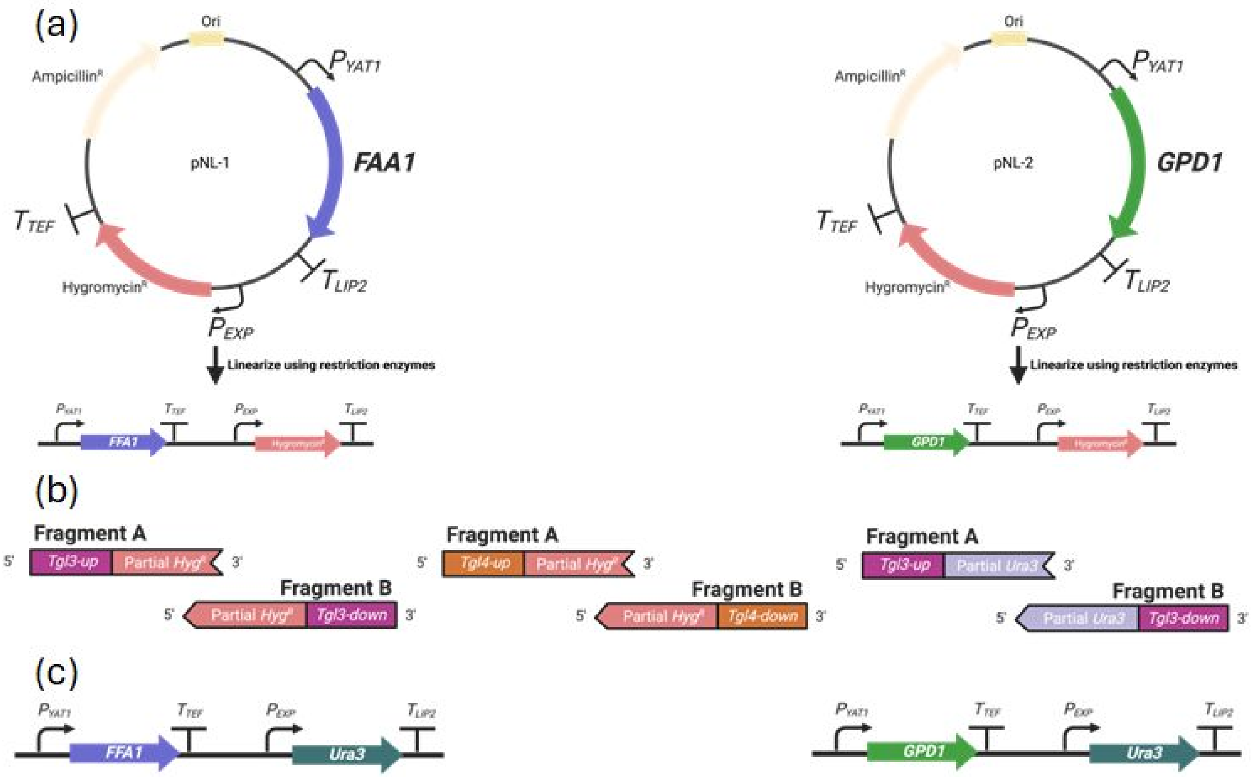

Figure S2. Plasmids, linear DNA, and DNA fragments used in this study to generate new engineered strains from Y8412. (a) Strain Y8412F+ and Y8412G+ were generated by linearization of these plasmids by restriction enzymes cutting and transformation into *Y. lipolytica* Y8412. (b) Construction of linear knockout cassettes for *TGL3* and *TGL4* by inserting a hygromycin-resistance gene into the *TGL3* and *TGL4* genes, respectively, using the split selection marker method and a *TGL3* knockout cassette with the *ura3* gene for knocking out *TGL3* in Y8412U$^-$. (c) Construction of linear *FAA1* and *GPD1* expression cassettes with the Ura3 gene for random insertion into Y8412T4$^-$U$^-$ to generate Y8412F$^+$T4$^-$ and Y8412G$^+$T4$^-$.

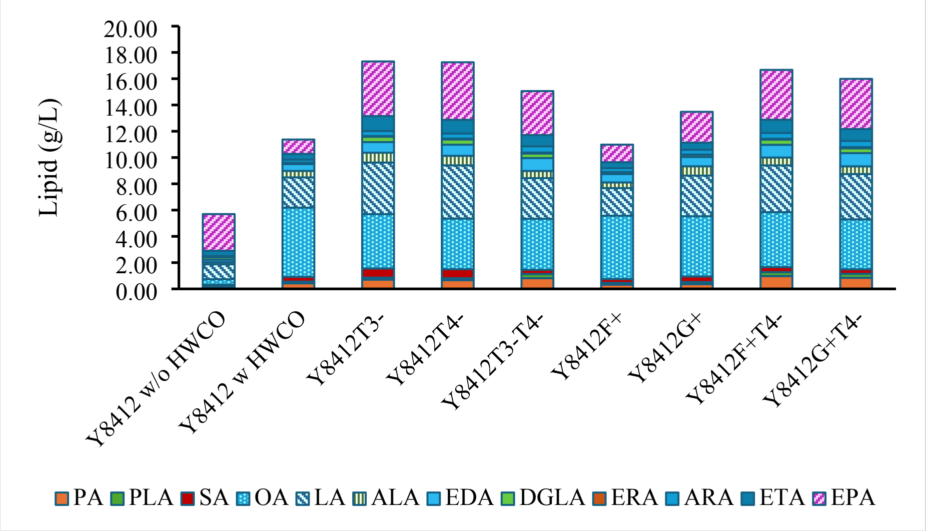

Figure S3. The titers of individual fatty acids produced byY8412 and engineered strains. Except strain Y8412, which was tested in flask fermentation with feeding of glucose only or cofeeding with glucose and hydrolyzed waste cooking oil (HWCO), all other engineered strain were tested in flask fermentation with cofeeding of glucose and HWCO. **Abbreviation**: PA, palmitic acid (C16:0); PLA, palmitoleic acid (C16:1); SA, stearic acid (C18:0); OA, oleic acid (C18:1); LA, linoleic acid (C18:2); ALA, alpha-linolenic acid (C18:3); EDA, eicosadienoic acid (C20:2); DGLA, dihomo-γ-linolenic acid (C20:3); ERA, eicosatrienoic acid (C20:3); ARA, arachidonic acid (C20:4); ETA, eicosatetraenoic acid (C20:4); EPA, eicosapentaenoic acid (C20:5).

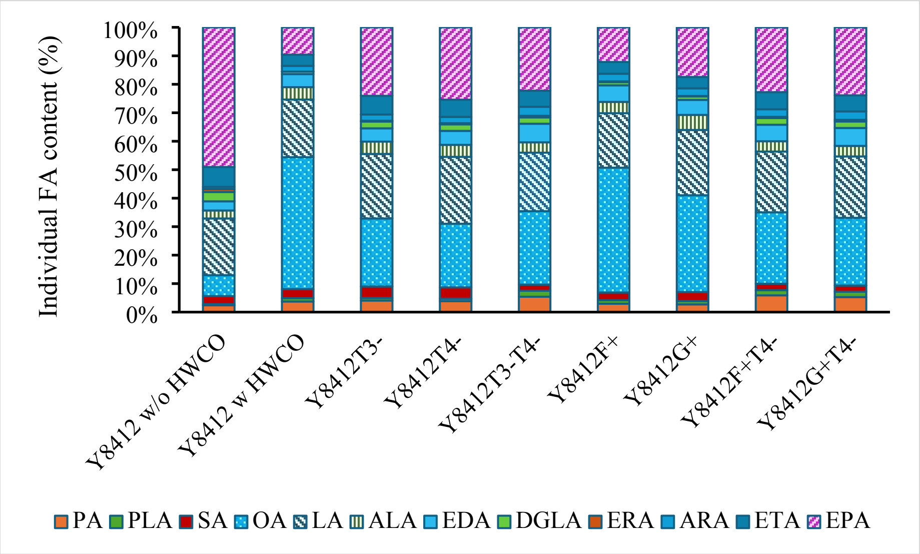

Figure S4. The fatty acid profile in lipid produced by Y8412 and engineered strains. Except strain Y8412, which was tested in flask fermentation with feeding of glucose only or cofeeding with glucose and hydrolyzed waste cooking oil (HWCO), all other engineered strain were tested in flask fermentation with cofeeding of glucose and HWCO. **Abbreviation**: PA, palmitic acid (C16:0); PLA, palmitoleic acid (C16:1); SA, stearic acid (C18:0); OA, oleic acid (C18:1); LA, linoleic acid (C18:2); ALA, alpha-linolenic acid (C18:3); EDA, eicosadienoic acid (C20:2); DGLA, dihomo-γ-linolenic acid (C20:3); ERA, eicosatrienoic acid (C20:3); ARA, arachidonic acid (C20:4); ETA, eicosatetraenoic acid (C20:4); EPA, eicosapentaenoic acid (C20:5).

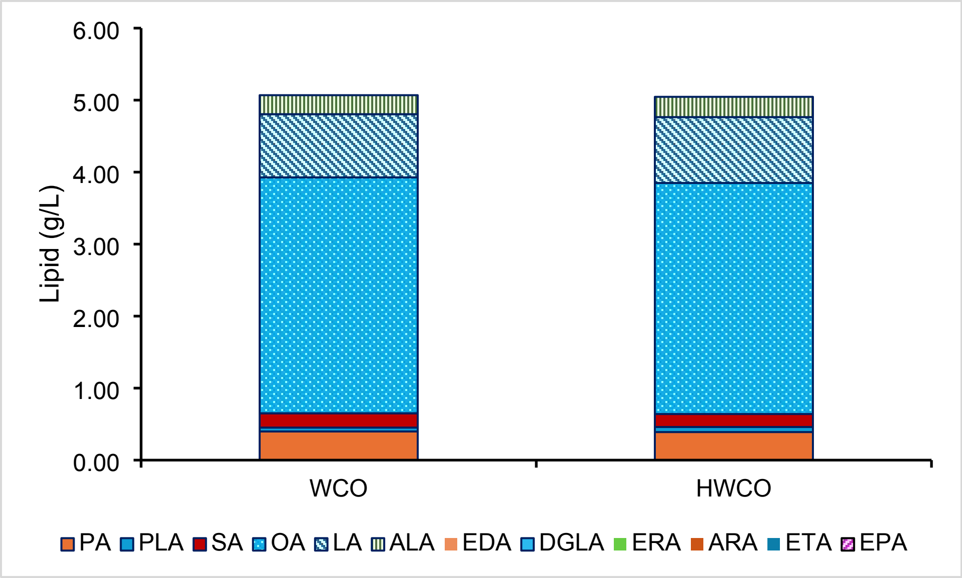

Figure S5. The mass of each fatty acid in 5g WCO and HWCO was analyzed by GC-FID. **Abbreviation**: PA, palmitic acid (C16:0); PLA, palmitoleic acid (C16:1); SA, stearic acid (C18:0); OA, oleic acid (C18:1); LA, linoleic acid (C18:2); ALA, alpha-linolenic acid (C18:3); EDA, eicosadienoic acid (C20:2); DGLA, dihomo-γ-linolenic acid (C20:3); ERA, eicosatrienoic acid (C20:3); ARA, arachidonic acid (C20:4); ETA, eicosatetraenoic acid (C20:4); EPA, eicosapentaenoic acid (C20:5).

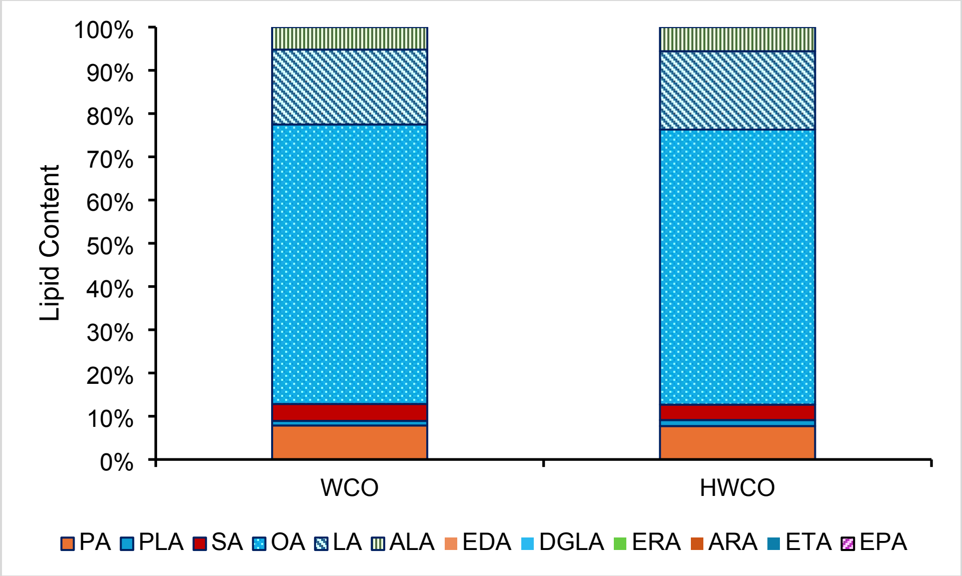

Figure S6. The fatty acid profile of WCO and HWCO analyzed by GC-FID. **Abbreviation**: PA, palmitic acid (C16:0); PLA, palmitoleic acid (C16:1); SA, stearic acid (C18:0); OA, oleic acid (C18:1); LA, linoleic acid (C18:2); ALA, alpha-linolenic acid (C18:3); EDA, eicosadienoic acid (C20:2); DGLA, dihomo-γ-linolenic acid (C20:3); ERA, eicosatrienoic acid (C20:3); ARA, arachidonic acid (C20:4); ETA, eicosatetraenoic acid (C20:4); EPA, eicosapentaenoic acid (C20:5).

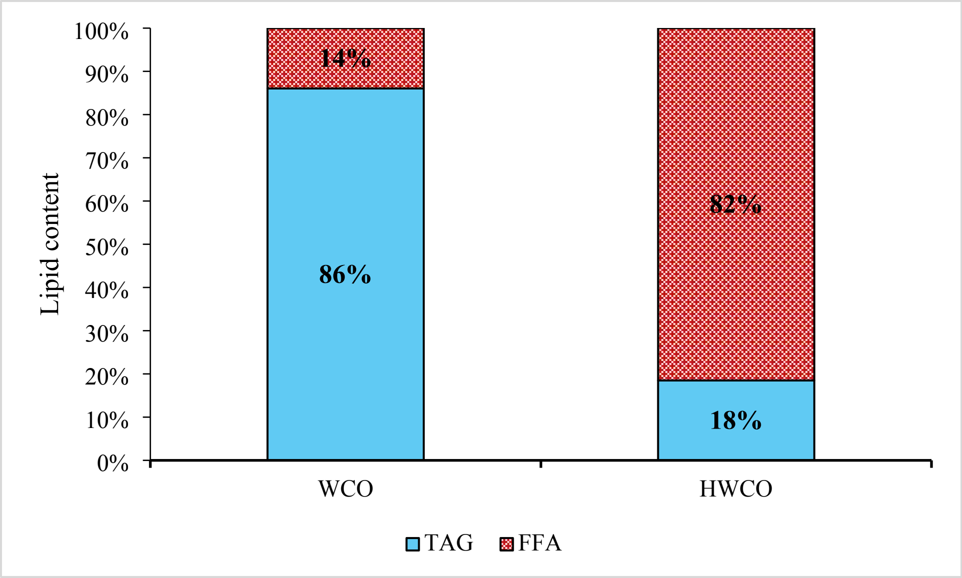

Figure S7. GC-FID Analysis results for FFAs and TAGs content in the original WCO and HWCO used in this study.

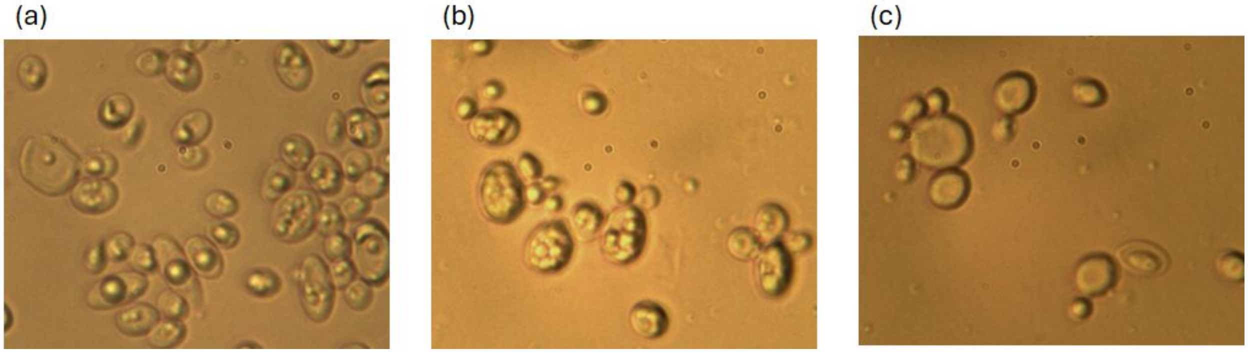

Figure S8. 1000x microscope picture of *Yarrowia lipolytica* Y8412 and Y8412T4^{-} cells from the end of the fed-batch flask cultures (t = 144 hr). (a) Y8412 fed with glucose, (b) Y8412 fed with glucose with HWCO, (c) Y8412T4^{-} fed with glucose with HWCO.

Table S1. Primers used in this study.

Name	Sequence
pFAA1_FP	ggagaagaaggtgcccaaggaatgg
pFAA1_pCfB6574_RP	ggttgattccgaacagaaggaatgcacgcgGGATCCctaagactgctcgtagcactcatc
pFAA1_RP	tcgtaggcagtgacaatgggaatac
pFAA1_YAT1_FP	CATTCTCACCACCCTAATTCACAAGGATCCatggtcggatacacaatttcctcaaagccc
pYAT1_FAA1_RP	gggctttgaggaaattgtgtatccgaccatGGATCCTTGTGAATTAGGGTGGTGAGAATG
pYAT1_GPD1_RP	acgcagggacgatcgaagtagagcgctcatGGATCCTTGTGAATTAGGGTGGTGAGAATG
pYAT1_pCfB6574_FP	ccacacttcaacggaatgcgtgcgGGATCCATAAGTTTGCAAAAAGATCGTATTATAGTT
pYAT1_pCfB6574_GPD1_FP	ttccgaacagaaggaatgcacgcgGGATCCATAAGTTTGCAAAAAGATCGTATTATAGTT
pYAT1_YAT1_FP	ATAAGTTTGCAAAAAGATCGTATTGGATCCATAAGTTTGCAAAAAGATCGTATTATAGTT
Amp-R	ATAATACCGCGCCACATAGC
Kan-R	ATCGCGAGCCCATTTATACC
220718FAA1_Ptef_R	ggactggctgttgtatttcgacac
220718U_FAA1_C.F	GGACGCCGTCTGTTCCAACAAAAA
220718U_FAA1_C.R	tccatatccccatccctctccaca
20220620_Tgl3_SU_R	TCAACAGTTCGGTACTGCTGTCCG
20220620FAA1_F	ccctgtgttgaatccatccatctt
20220620FAA1_F2	cgtGCGATCGCATCGATATAAGTT
20220620FAA1_Ptef_F	AGAAGATTAACTGTTAGccccacgttgccggtctt
20220620FAA1_Ptef_R2	cggaggccactagtggatctgata
20220620FAA1_R	GTAGGAGGGCATtgctgtagatatgtcttgtgtgtaaggg
20220620FAA1_Ura3_F	acaagacatatctacagcaATGCCCTCCTACGAAGCTCGA
20220620FAA1_Ura3_R	gcaacgtggggCTAACAGTTAATCTTCTGGTAAGCCTCCc
20220620Ura3_GPD1_F	ctgagaacagctgaagcttcgtac
20220620Ura3_GPD1_F2	catctgcgttgaacattgagaccc

pGPD1_YAT1_ FP	CATTCTCACCACCCTAATTCACAAGGATCCatgagcgctctacttcgatcgtccctgcgt
pYAT1_FAA1_RP	gggctttgaggaaattgtgtatccgaccatGGATCCTTGTGAATTAGGGTGGTGAGAATG
pYAT1_GPD1_RP	acgcagggacgatcgaagtagagcgctcatGGATCCTTGTGAATTAGGGTGGTGAGAATG
pYAT1_pCfB6574_FP	ccacacttcaacggaatgcgtgcgGGATCCATAAGTTTGCAAAAAGATCGTATTATAGTT
pYAT1_pCfB6574_GPD1_FP	aacagaaggaatgcacgcgatcgcGGATCCATAAGTTTGCAAAAAGATCGTATTATAGTT
pYAT1_YAT1_RP	AACTATAATACGATCTTTTTGCAAACTTATGGATCCacgcgatcgcGGATCCATAAGTTT
220718U-C.F	CAGCAGTACCGAACTGTTGAGGAT
220718U_GPD1_C.R	GGTTGACGGCGGACATGTGTTTAA
20220620GPD1_F	gcctccggattccgaacagaaggaatgcacg
20220620GPD1_R	aaaccagctgtcccattgacagaa
20220620GPD1_R2	gcagaggatagtgcttacgtgcaa
20220620Ptef_R2	ccttctgttcggaatccggaggccactagtggatctgata
20220620Ura3_GPD1_F	ctgagaacagctgaagcttcgtac
20220620Ura3_GPD1_F2	catctgcgttgaacattgagaccc
3_KO_down.R	GGTACAGAGTTGCTGCTCAAGCTA
3_KO_down.R2	ATGGGCATCAACACCGTTACAATC
3_KO_down2.FOR	tcagctgttctcaGACCCCGAGCAGAGCACATAACTA
3_KO_up.F	GTTTGCGAGAGAGCGACAAGAGAG
3_KO_up.F2	TCGCACAGGATATCCACAAGTTCG
3_KO_up2.REV	tggcctccgAATCGCGGTAGAAAAGGAGCTgaa
1013Tgl3_down_C.R	AGCCCAGACTTCTGATATCACCAG
1013Tgl3_up_C.F	TCCTGTTGTCAAGTTGTTCAGCCC
220118Hyg.F1	ggtttccactatcggcgagtactt
220118Hyg.R1	cgacgtctgtcgagaagtttctga
220118hyg_check_F	aataggtcaggctctcgctgaatt
220118hyg_check_R	aattcagcgagagcctgacctatt
HygR_C.R	tcagagcttggttgacggcaattt

HygR3.FOR	TGCTCGGGGTCtgagaacagctgaagcttcgtacg
HygR3.REV	ttttctACCGCGATTcggaggccactagtggatctgata
4_KO_down.R	GACAAAGTTGATGGGGTGGGAGAA
4_KO_down.R2	CAGTTCCCACCAAGGTCAGAAGAG
4_KO_down2.FOR	gctgttctcagCTGTGGCATGGCTAACGTTGAtat
4_KO_up.F	ATCGTACTCGTACCGGTTCTATGC
4_KO_up.F2	CGGAAGATGGCATATATACGACCG
4_KO_up2.REV	ctagtggcctccgATGCATGTACACACTGTAGGGAGG
1013Tgl4_down_C.R	TTGTCAGCGCCCTTGATGAGAATT
1013Tgl4_up_C.F	TCCAGAACATGATCAAGTGGCAGC
HygR4.FOR	CCATGCCACAGctgagaacagctgaagcttcgtac
HygR4.REV	GTGTACATGCATcggaggccactagtggatctgata
220824Tgl3_D.F	tgttctcagGACTGGCGGAGGAAAACAAACACA
220824Tgl3_D.R	CACCAAGGTGAACAACAGCACCAA
220824Tgl3_D.R2	ATGTCGTTGAGGGTGTACTCGATT
220824Tgl3_U.F2	CCGCATCCAGTAGAGAGAGTGAAG
220824Tgl3_U.R	agtggcctccgACTCGATAAAGTCTCTCAGACGGC
220824Tgl3_Ura3.F	GACTTTATCGAGTcggaggccactagtggatctgata
220824Tgl3_Ura3.R	tttcCTCCGCCAGTCctgagaacagctgaagcttcgtac
